# Staining of cerebellar cortex granular layer interneurons with natural dye of Madder

**DOI:** 10.1186/s40673-016-0050-6

**Published:** 2016-06-02

**Authors:** Anneh Mohammad Gharravi

**Affiliations:** School of Medicine, Shahroud University of Medical Sciences, Shahroud, Iran

**Keywords:** Madder, Cerebellum, Unipolar brush cells, Lugaro cell, Golgi neurons

## Abstract

**Background:**

The objective of the present study was an investigation of root Rubia Tinctorum (Madder) as a natural dye to identification of granular layer interneurons of the rat cerebellum.

**Methods:**

Seven to ten micrometre sections were collected from the cerebellum and stained only with Madder for 2, 24 and 48 h. Other sections were stained with Madder then with hematoxyllin, cresyl violet, eosin, light green. Microscopic identification of cells was performed based on cell morphology, reaction and binding of with the dye. All data were expressed as mean ± SD in and significance was set at *p* ≤0.05.

**Results:**

Madder with alum as mordant resulted a deep red staining of interneurons. Unipolar brush cells (UBCs) were observed with a cell body diameter intermediate between granule and Golgi cells in the superficial layer of the granular layer. Golgi cells were identified almost as large as Purkinje cells with irregular rounded or polygonal morphology. Lugaro cells were observed as spindle-shaped cells adjacent to Purkinje layer.

**Conclusion:**

Results of the present study showed that mader could stain granular layer interneurons in cerebellum cortex of rat.

## Background

Histologically, the cerebellar cortex is divided into three layers: the molecular, the Purkinje and the granular layers. There are three distinct interneurons in the granular layer; Golgi, Lugaro and UBCs [1, 2]. UBCs involve in critical function such as the sensorimotor processes that regulate body, head and eye position [3]. The cells are a unique type excitatory, glutamatergic interneurons that receive direct mossy fiber excitation, play a role in amplifying mossy fiber signals [4]. UBCs are intermediate in size between granule cells are found in abundance within the granule cell layer, particularly in the uvula-nodulus [5]. Golgi cells are inhibitory (GABAergic) interneurons and sample mossy fiber activity, provide both feedforward and feedback inhibition of granule cells. The cells are the largest and most numerous interneurons within the granular layer [6, 7]. Granule cells receive glutamategic inputs from mossy fibers and UBCs, and glycinergic/GABA aergic inputs from Golgi cells. Lugaro cells have numerous axosomatic and axodendritic contacts with all cerebellar cortical neurons and fibers and they function as inhibitory interneurons. These spindle-shaped cells locate just underneath the Purkinje cell layer are major effectors for serotonin in the cerebellar cortex [2, 8]. Despite the accumulating evidence pointing to a key role of interneurons in granular layer processing, still it is not clear how they operate.

Morphological identification of the interneurons performed by histochemistry methods also based on their neurotransmitter contents [3, 6, 8]. These synthetic dyes are hazardous in nature and are not environment friendly and are risky to human and animal health. Nowadays there is a worldwide concern in the use of eco-friendly and biodegradable materials. Alsot, these synthetic dyes are expensive. Therefore, the use of cheaper, naturally occurring dyes is being viewed as an alternative to synthetic dyes [9]. One of the most commonly used dye in histology; hematoxyllin is a natural dye. Natural dyes come from roots, flowers, leaves, fruits and barks of plants. The root of Mdder, well known as a source of the anthraquinone, alizarin has background staining ability for neuroglia, spinal cord is cheaper and bio-friendly [10].

Differences in neurohistology phenotype could provide the basis for distinct identification of interneurons. Based on the aforementioned, objective of the present study was investigation of root Madder as a natural dye with potential histopathological application to identification of granular layer interneurons.

## Methods

### Madder root preparation and staining solutions

Fresh roots of Madder collected from Gorgan province- north of Iran, cut into tiny bits of about 1 mm in diameter and dried in an open air and a very fine powder obtained. 5 g of the powder dissolved in 100 ml of water and iron alum. The mixtures boiled in a water bath for 5 min to completely dissolve the solutes, and obtain bright red dye. After a pilot study, we used alum as a mordant, which will give a deep red. If a copper dye vat is used the color will be brighter.

Then the solution placed in dark jar. Two solutions; basic (an initial solution that has pH over 7) and acidic pH 4–5 (by adding acid acetic to initially solve) prepared.

### Staining design

Seven to ten micrometre sections were collected from the cerebellum of Wistar rats (6 to 11 weeks of age). After fixation with Bouin’s fluid for 24 h, the cerebellums were transferred to 70 % alcohol, dehydrated through ascending grades of alcohol. Then the cerebellums were cleared in xylene and embedded in paraffin wax and cut. Sections of cerebellums divided into 9 groups. Sections of groups 1, 2 and 3 were stained only with Madder for 2, 24 and 48 h respectively. Sections of groups 4, 5,6 and 7 were stained first with with Madder then hematoxyllin, cresyl violet, eosin and light green respectively.

Sections of group 8 were stained by hematoxyllin and eosin based on the Mayer method (16). And group 9 were stained with cresyl fast violet staining.

Briefly, staining procedure for groups 1, 2 and 3 were as follows:

1. Deparaffination of sections and hydration of water
2. Staining withMadder for, 24 and 48 h respectively
3. Washing in running tap water for 5 min
4. Dehydration in 95 % and absolute alcohols, two changes of 2 min each
5. Clearing in Xylene, −Mounting

For groups 4, 5, 6 and 7 the procedures were continued to step 3 and sections stained with hematoxyllin (3 min), cresyl violet (1 min), eosin (3 min) and light green (1 min) respectively.

Microscopic identification of cells was performed based on cell morphology, reaction and binding of cells with the dye.

Statistical analysis was made with SPSS V.22 and the variables were tested using the Paired Samples Test. All data were expressed as mean ± SD in each group and significance was set at *p* ≤0.05.

## Results

Our results indicate that madder with alum as mordant result a deep red staining of granular layer interneurons observed in acidic solution of madder. But the basic solution of madder resulted a poor and ambiguous staining.

**Fig. 1:**
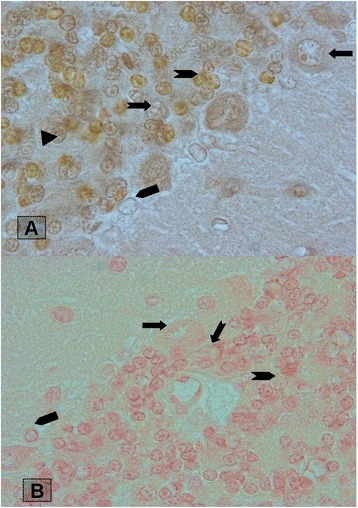
Section of cerebellum showing granular layer interneurons stained with different methods. Upper should be changed into **a**: Stained section with acidic Madder for 2 h and Bottom should be changed into **b**: with Madder for 24 h. Magnification 200×

**Fig. 2:**
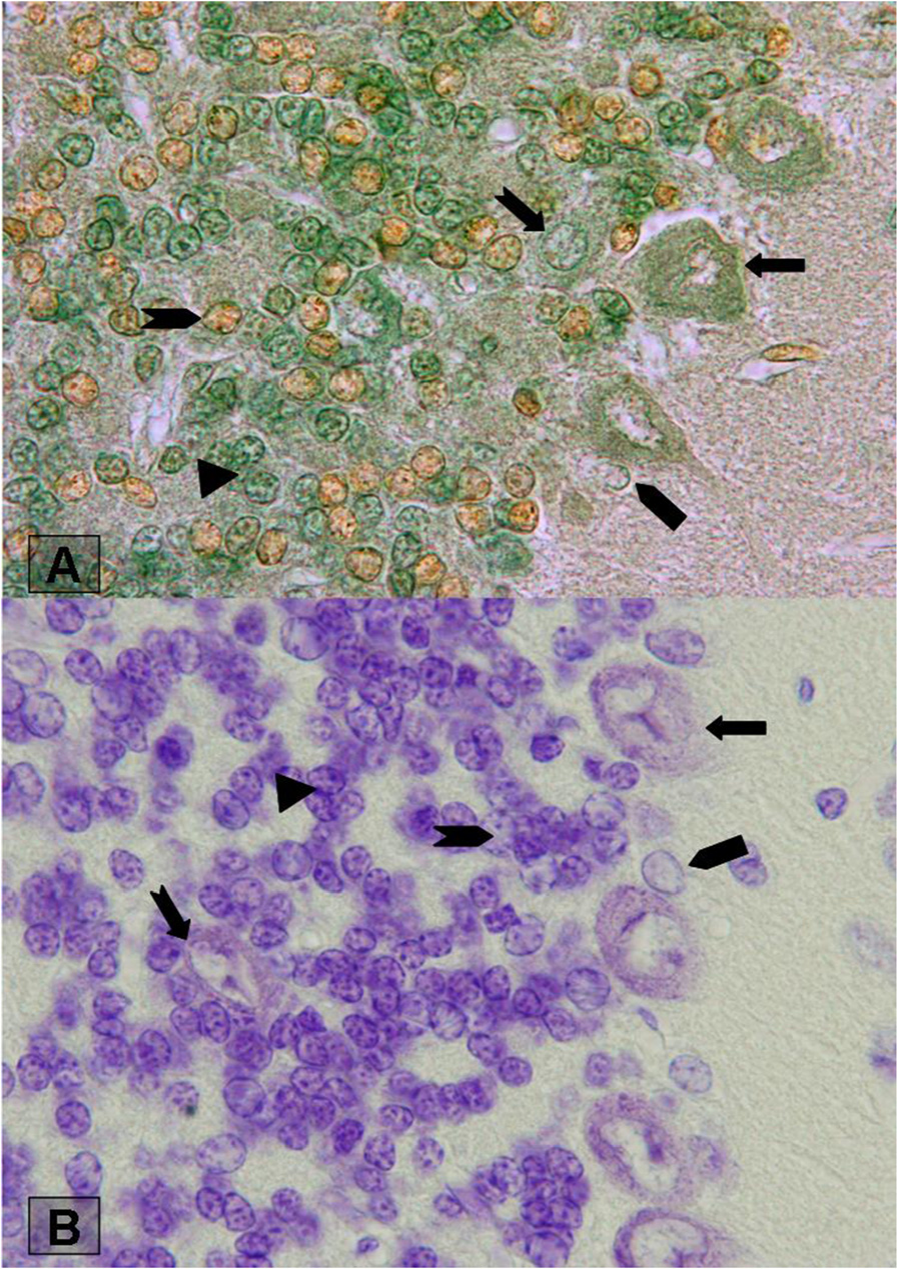
Section of cerebellum showing granular layer interneurons. Upper should be changed into**a**: Stained section with acidic Madder & light green, bottom should be change into **b**: Madder with cresyl fast violet. Magnification 200×

We tested nine solutions, and only groups 1, 2 and 7 of those were acceptable for consistent staining. Staining by solutions of other groups result negative reaction into interneurons. Morphological characteristics of groups 1, 2 and 7 when compared with cresyl violet staining depicted in Table 1, Figs. 1 and 2.

**Table 1:** Morphological characteristics of interneuron’s granular layer of rat cerebellum which stained with madder

	Madder- 2 h		Madder-24 h		Madder –fast green		Cresyl fast violet	
	Nuclei shape	Cytoplasm	Nuclei shape	Cytoplasm	Nuclei shape	Cytoplasm	Nuclei shape	Cytoplasm
Purkinje	Round	brown	Oval	Pale red	Oval-round	Pale green	Oval-round	Pale blue
Golgi	Oval	brown	Oval	Pale red	Round-oval	green	Oval-round	Pale blue
Lugaro	fusiform	brown	fusiform	Pale red	fusiform	Pale green	fusiform	blue
Unipolar brush cell	Round-oval	brown	Round-oval	Pale red	oval	yellow	round	Pale blue
Granule	Round-oval	brown	Round-oval	Pale red	round	Pale green	round	blue

As shown in figures, UBCs were observed with a cell body diameter intermediate between granule and Golgi cells. The nuclei of UBCs were deeply indented and showed little condensed chromatin and predominantly dispersed chromatin. Moreover, the cytoplasm contained numerous large dense core vesicles, and relatively little granular endoplasmic reticulum, whereas the granule cell nucleus contained abundant heterochromatin. Moreover, UBCs were observed in the superficial layer of the granular layer (Fig. 2). Golgi cells were identified almost as large as Purkinje cells with irregularly rounded or polygonal morphology (Fig. 2). The cell bodies of Golgi cells dispersed throughout the granular layer with predominant nucleoli. However, similar to The UBCs, Golgi nuclei were deeply indented and showed little condensed chromatin and predominantly dispersed chromatin.

Lugaro cells were observed as spindle-shaped cells adjacent to Purkinje layer. Group 1 gave the best result of identification of these cells. The cytoplasm was pale with nucleus.

**Fig. 3:**
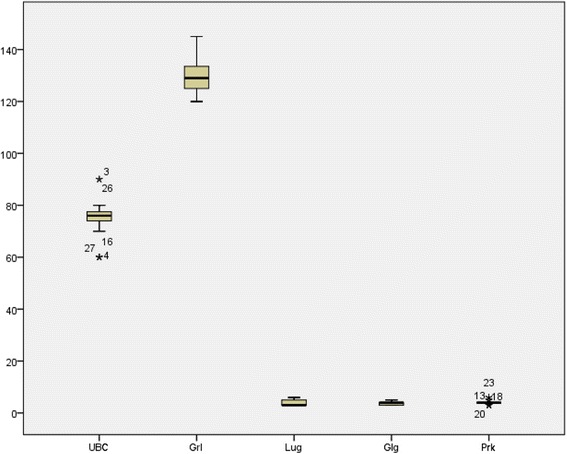
Comparing of cell in 500 μm area section of cerebellum. Lug:Lugaro, Glg:Golgi, Prk:purkinj, UBS:UBCs and Grl:granule

Mean ± SD of Lugaro, Golgi, purkinj, UBCs and granule cell were 3.87 ± 1.056, 3.77 ± .717, 4.03 ± .795, 74.94 ± 6.678 and 130 ± 6.792 respictevely. UBCs distributed significantly in granular layer of cerebellum (p = 000) (Fig. 3).

## Discussion

There are several reports that synthetic dyes cause allergic-like symptoms or are carcinogenic. Therefore, to avoid some hazardous synthetic dyes to humans and animals, the cheaper, eco-friendly and biodegradable natural dyes from various parts of plants have been currently more interested for histological staining purposes [11].

Results of the present study indicate that madder with alum as mordant result a deep red staining of granular layer interneurons. UBCs were observed with a cell body diameter intermediate between granule and Golgi cells in the superficial layer of the granular layer, adjacent to the Purkinje cell layer. The cell bodies of Golgi cells dispersed throughout the granular layer with predominant nucleoli. Lugaro cells were observed as spindle-shaped cells adjacent to Purkinje layer. Interestingly, many UBCs compared to granule cells which are the most abundant cells in the cerebellar cortex were appeard with madder staining. Altman and Bayer (1997) reported that on average, rat UBCs outnumber Golgi cell by a factor of 3 or more and approximately equal the Purkinje cells [12]. But, according to the literature, there is a need for quantitative analyses of S distribution after total labeling of the cell population and after differential labeling of the UBCs subclasses.

Madder contains six dyes in the form of their glycosides, namely alizarin, Xanthopurpurin, xanthopurpurin-3-carboxylic acid, Rubiadin, Purpurin and Pseudopurpurin [13]. Pseudopurpurin and xanthopurpurin yields the orange and the yellow dyes respectively [13]. Furthermore, Alizarin has been used to evaluate bone tissue and calcium-rich deposits by cells in culture [14]. There are some studies that indicate staining of neuroglia, mitochondria and spinal cord with Alizarin [15]. Recently, histological staining of animal tissue using Madder extracts as stains has been reported by Deepali et al. [16]. Also staining human lymphocytes experiments with madder root has been performed by Cücer et al. [17].

Many factors such as application of mordant, temperature, pH of dye solution and time can influence staining potency. Salt or hydroxide of divalent or trivalent metals which forming a bridge between the tissue and the dye are known as a mordant. In the present study, mordant was necessary for madder which staining to occur. Therefore, a tissue mordant-dye complex is formed [18]. Moreover, although some dyes such as phoshotungstic acid hematoxyllin method for nervous tissue takes place at a high temperature, in the present study, Madder staining properties was not temperature dependent. But, Madder staining is pH dependent and as the cationic or basic dye showed an affinity for acidic constituents of the tissue such as nuclei.

## Conclusions

Madder in the form of their glycosides could stain granular layer interneurons (Golgi, Lugaro and UBC) in cerebellum cortex of rat. The staining property was pH dependent. Madder with alum mordant as the cationic or basic dye showed an affinity for acidic constituents of the tissue such as nuclei.
